# Towards identification of molecular mechanisms of short stature

**DOI:** 10.1186/1687-9856-2013-19

**Published:** 2013-11-20

**Authors:** Lindsey A Waldman, Dennis J Chia

**Affiliations:** 1 Institutional addresses: Division of Pediatric Endocrinology & Diabetes, Department of Pediatrics, Icahn School of Medicine at Mount Sinai, One Gustave L. Levy Place, New York, NY 10029, USA

**Keywords:** Short stature, Growth hormone, IGF-1, GWAS, Chromosomal microarray, Sequencing, Chromatin

## Abstract

Growth evaluations are among the most common referrals to pediatric endocrinologists. Although a number of pathologies, both primary endocrine and non-endocrine, can present with short stature, an estimated 80% of evaluations fail to identify a clear etiology, leaving a default designation of idiopathic short stature (ISS). As a group, several features among children with ISS are suggestive of pathophysiology of the GH–IGF-1 axis, including low serum levels of IGF-1 despite normal GH secretion. Candidate gene analysis of rare cases has demonstrated that severe mutations of genes of the GH–IGF-1 axis can present with a profound height phenotype, leading to speculation that a collection of mild mutations or polymorphisms of these genes can explain poor growth in a larger proportion of patients. Recent genome-wide association studies have identified ~180 genomic loci associated with height that together account for approximately 10% of height variation. With only modest representation of the GH–IGF-1 axis, there is little support for the long-held hypothesis that common genetic variants of the hormone pathway provide the molecular mechanism for poor growth in a substantial proportion of individuals. The height-associated common variants are not observed in the anticipated frequency in the shortest individuals, suggesting rare genetic factors with large effect are more plausible in this group. As we advance towards establishing a molecular mechanism for poor growth in a greater percentage of those currently labeled ISS, we highlight two strategies that will likely be offered with increasing frequency: (1) unbiased genetic technologies including array analysis for copy number variation and whole exome/genome sequencing and (2) epigenetic alterations of key genomic loci. Ultimately data from subsets with similar molecular etiologies may emerge that will allow tailored interventions to achieve the best clinical outcome.

## Introduction

Poor growth is among the most common reasons for referral to pediatric endocrinology specialists. The evaluation begins with a detailed history and physical examination. Past medical records indicating the growth pattern in infancy and childhood are highly desirable to shape the context of the presentation. The birth history should address any known history of intrauterine growth retardation (IUGR) and whether birth parameters indicate small for gestational age (SGA), as approximately 10-15% of SGA infants fail to display appropriate catch-up growth in the first years of life and do not reach an adult height in the normal range [1]. Poor weight gain in excess of poor linear growth better fits considerations for failure to thrive and carries a largely distinct differential diagnosis. The physical examination may reveal clues to an underlying etiology, such as a goiter with hypothyroidism, and should include assessment for abnormal body proportions that may be indicative of a skeletal dysplasia.

In the absence of specific features identified in the history and physical, a laboratory evaluation that assesses for pathologies that characteristically lead to poor growth is typically initiated (Table 1). Bone age x-rays can provide a framework for adult height prediction; however, they do not reliably distinguish between normal and pathologic growth patterns. Additional testing is guided by clinical suspicion and not typically performed in a screening matter. For example, growth failure accompanied by excessive weight gain prompts an evaluation for Cushing syndrome, or characteristic facies or heart murmur may warrant an investigation for Noonan syndrome. Similarly, genetic testing for abnormalities of the *SHOX* gene is usually reserved for those with evidence of a skeletal dysplasia, most characteristically Madelung deformity, or a highly suggestive inheritance pattern [2].

**Table 1:** Etiologies for short stature and common screening tests

**Etiology**	**Screening tests**
**Genetic short stature**	
Chromosomal aneuploidy, including Turner syndrome	Karyotype in girls
Microdeletion or duplication	
Single gene disorders	
Polygenic	
**Constitutional delay of growth and puberty**	
**Hormonal pathologies**	
Growth hormone deficiency	IGF-1, IGF BP-3
Hypothyroidism	Thyroid function tests
Cushing syndrome	
**Systemic diseases**	
Inflammatory disease, e.g. IBD, JIA	erythrocyte sedimentation rate
Celiac disease	tissue transglutaminase antibodies
Renal disorders	electrolytes, creatinine
Liver disorders	liver function tests
Hematologic disorders	complete blood count
**Birth history of IUGR/SGA**	
**Medications**	

### Investigations to identify an etiology for short stature are frequently unrevealing

A review prepared by international growth experts estimated that approximately 80% of short children evaluated by pediatric endocrinologists do not have an identified etiology and are therefore classified as idiopathic short stature (ISS) [3]. A history of SGA is found in ~15% of short children, thereby making it the single most common identified etiology, although it is perhaps better termed an *association* as the mechanisms for absence of catch up growth and persistent short stature in a small percentage of those with a history of SGA remain largely unknown. Data accumulated from multiple studies finds that only ~5% of short children have a pathologic laboratory finding identified by routine screening [4-6]. Based on the low yield and costs of these screening tests, the rationale for routinely performing them in otherwise asymptomatic short children has been questioned [7].

While the term ISS is extensively used in the literature and is accepted clinically as an indication for the use of GH by the FDA, the concept has many limitations that detract from its utility. Although adult height is a classic continuous phenotype determined by the interaction of multiple genetic, epigenetic, and environmental factors, designating an etiology implies that there is a single root cause for short stature in any individual subject. This admittedly flawed assumption likely carries greater validity when reserved for those with a more severe phenotype, e.g. height SDS < -3 or < -4, rather than those at the lower limit of the normal range, and we therefore strongly prefer restricting the term ISS for those with more profound short stature. ISS specifically does not exclude elements of familial short stature (FSS) and constitutional delay of growth and puberty (CDGP) [3], which have long been recognized as among the most common reasons for being short for age but are likewise difficult to classify as normal variants or pathologic in any individual. Twin studies have revealed the heritability of height to be in the 80-90% range [8], and calculating mid-parental height alone can explain 40% of variation in adult height [9]. The strong genetic component of height fuels aspirations to identify molecular mechanisms of short stature in individual patients, rather than simply label the cause as idiopathic.

### Interpretation of the GH–IGF-1 axis in ISS can be challenging

The GH–IGF-1 axis is the most important hormonal axis governing growth, and therefore any evaluation of a child presenting with poor growth must include consideration for potential pathologies that impact GH and IGF-1 (Table 2) [10]. The challenge faced by pediatric endocrinologists arises from the interpretations of laboratory values of this axis, particularly given the spectrum of ranges observed in the context of FSS and CDGP. As an illustration of the difficulties in interpreting the laboratory values, the ISS consensus statement concluded that GH status should not be considered strictly as GH deficient or GH sufficient, but rather a range of probabilities spanning 0-100% [11].

**Table 2:** GH Research Society Consensus Guidelines [10]^*^, criteria for investigations of the GH-IGF axis

**Criteria**	
●	Severe short stature, height < -3 SDS
**●**	Height <1.5 SDS below midparental target height
**●**	Height < -2 SDS AND height velocity < -1 SDS for chronological age for >1 year; decrease in height SDS of >0.5 SDS over 1 year (children >2 years)
**●**	Height velocity < -2 SDS for >1 year, or height velocity < -1.5 SDS for 2 years
**●**	Signs indicative of an intracranial lesion
**●**	Signs of multiple pituitary hormone deficiency
**●**	Neonatal signs and symptoms of GH deficiency

^*^endorsed by the Councils and Drug and Therapeutics Committees of the European Society for Pediatric Endocrinology and the (formerly Lawson Wilkins) Pediatric Endocrine Society, the Australasian Pediatric Endocrinology Group, the Japanese Society for Pediatric Endocrinology, and the Sociedad Latinoamericana de Endocrinologia Pediatrica.

Serum IGF-1 is produced predominantly by the liver in response to GH, and has good reproducibility when assayed in reference laboratories [12]. With few exceptions [13], an IGF-1 value that is in the upper half of normal range for age has high negative predictive value for GH deficiency [10]. IGF-1 values vary considerably in otherwise normal children due to several factors including age, pubertal stage, and nutritional status. As children presenting for growth evaluations commonly have delayed puberty (or delayed bone age findings in children of prepubertal age), it is not entirely surprising that IGF-1 levels are frequently low for chronologic age in children with ISS, with reports of IGF-1 values < -2 SDS for chronologic age ranging from 25-50% [14-16]. Short children with low IGF-1 levels most often proceed with stimulation testing using two provocative agents as the gold-standard assessment of GH secretion, with a peak GH of <10 μg/L traditionally used as the cutoff to define GH deficiency [10]. Still, it is well acknowledged that GH stimulation testing is problematic [17]. There are no clear data to establish how a normal GH response is defined, and the distinction between isolated partial GH deficiency and ISS has been labeled “largely arbitrary” [11].

Pediatric endocrinologists have inherent biases in focusing on pathologies of the GH–IGF-1 axis to explain poor growth, and several patterns of the GH–IGF-1 axis in ISS evoke potential pathologies. The high prevalence of low IGF-1 levels in ISS has been discussed, and when coupled with normal stimulation testing can be termed primary IGF deficiency. GH doses necessary to achieve normalization of IGF-1 are higher (with a broad range) in ISS than GH deficiency [18,19], consistent with partial GH resistance. Furthermore, the growth velocity in response to achieving a similar IGF-1 level is also less in ISS than GH deficiency [19], consistent with partial IGF resistance. Measurements of GH binding protein (GHBP), commonly considered a surrogate for GH receptor expression, are low in approximately 90% of children with ISS [20]. Despite these combined features, it is the rare subject in whom the laboratory findings and molecular genetic studies establish a defined pathological etiology of the GH–IGF-I axis, outside of GH deficiency.

### Single gene mutations of genes of the GH–IGF-1 axis can present with short stature

**Figure 1:**
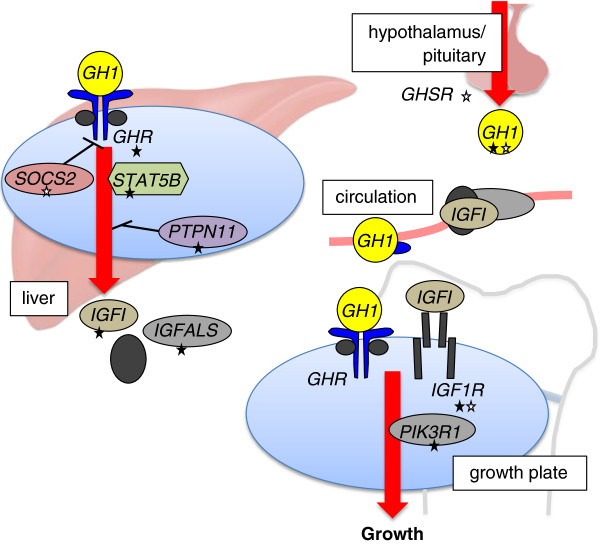
**Described genetic defects of the GH–IGF-1 axis associated with growth.** Multiple genes of the GH–IGF-1 axis have been identified that impact growth, in the setting of both case reports with severe mutations producing a profound phenotype (*filled stars*) and common variants that contribute a small effect in height GWA studies (*clear stars*). Note that there is modest overlap in the two sets of genes, with only *GH1* and *IGF1R* implicated in both sets. The list of single gene defects functioning at the level of the hypothalamus and pituitary that give rise to GH deficiency has been abbreviated in the figure for clarity.

Analogous to other hormone systems, defects in multiple steps of the GH–IGF-1 axis have long been hypothesized as mechanisms of pathophysiology (Figure 1). Laron first described a cohort of 3 children who clinically resembled those with GH deficiency but had elevated levels of GH by laboratory testing [21]. With the cloning of the gene for the GH receptor in 1987 [22], autosomal recessive inherited defects of *GHR* were demonstrated as the first molecular etiology for the syndrome of GH insensitivity [23]. Since then, single gene defects in *IGF1*, *STAT5B*, *IGF1R*, and *IGFALS* have been identified that include a phenotype of short stature [24-28]. Other characteristic features that distinguish these cases include prenatal growth failure, microcephaly, and developmental delay with both *IGF1* and *IGF1R*[24,26], sensorineural deafness with *IGF1*[24], and immunodeficiency with *STAT5B*[25].

While these case reports have been instrumental in shaping understanding of genes necessary for normal growth, they do not establish that defects of these genes play a role in the poor growth in a substantial proportion of short children in the general population. A review from 2011 listed the total number of cases in the literature to be less than 300 [28], and even experienced pediatric endocrinologists may only have directly cared for a handful of patients known to carry such mutations among the thousands they have evaluated in their careers. With mutations of the *GHR*, *STAT5B*, *IGF1*, and *IGFALS* genes, homozygosity (generally only observed with parental consanguinity or a highly inbred population) or compound heterozygosity is characteristically necessary for the phenotype, supporting the notion that these cases only arise rarely. Heterozygous carriers of a mutation are described to have mild short stature that is at the lower end of the spectrum of the normal range [28,29], with one study of individuals with mutations of *IGFALS* indicating that a defective allele in the heterozygote state results in a loss of height of approximately 1.0 SDS [30]. In addition, two dominant-negative mutations of *GHR* have been described [31,32], and there have been recent reports of heterozygous mutations of *IGF1* associated with short stature [33,34]. It should be noted that individuals identified with mutations of *IGF1R* usually carry only a single defective allele [26,28], consistent with mouse models in which homozygous knockouts of the gene do not have sustained viability [35]. Finally, heterozygous gain-of-function mutations of *PTPN11*, which encodes the protein phosphatase SHP-2, are one genetic etiology of Noonan syndrome that has been suggested to function in altering the GH–IGF-1 axis [36-38]; however, the data demonstrating a distinction with other genetic etiologies of Noonan syndrome is not entirely conclusive [39,40].

Initial identification of individuals harboring these mutations understandably focused on those with a severe phenotype, whereas it has since been appreciated that there is a continuum of abnormalities with some relationship between genotype and phenotype. For example, *GHR* missense and nonsense mutations produce a more severe growth phenotype than dominant-negative and intronic pseudoexon mutations [28]. Goddard and co-workers reported that 8 of 100 children with ISS carried variants in *GHR*, leading to the conclusion that mutations of *GHR* are a relatively common explanation for poor growth; however, the absence of significant functional data coupled with the failure of the variants to predictably track with the stature phenotype in family members, particularly in the setting that 7 of 8 were observed in the heterozygous state with one normal allele, raises considerable doubt to their conclusion that the identified variants contribute substantially to partial GH insensitivity [41,42]. A common variant of *GHR* that lacks exon 3 has also been investigated for an association with height, with most studies only finding an effect on growth velocity with exogenous GH administration [43,44]. The absence of strong data notwithstanding, there is seemingly a steadfast perception that common genetic defects of the GH–IGF-1 axis underlie a significant proportion of poor growth.

### Height GWA Studies have provided insights to the biology of height

The common disease-common variant model holds that for complex polygenic traits and diseases, multiple common variants are present within the population that collectively confer an additive effect on the phenotype, and was first proposed to be applicable to height by the statistician and evolutionary geneticist Ronald Fisher nearly a century ago [45]. The most commonly assayed unit of genetic variation in the population is the single nucleotide polymorphism (SNP). Genome-wide association (GWA) studies seek to identify genetic loci that are associated with a particular phenotype in an unbiased manner by genotyping hundreds of thousands of SNPs simultaneously on a single microarray chip [46]. The investigator can then independently interrogate whether any individual SNP is associated with the phenotype of interest. Sample size in the neighborhood of tens of thousands is critical because multiple hypothesis testing necessitates stringent statistical thresholds to avoid false-positive results, particularly given that common SNPs characteristically have a small effect size [47]. Current commonly used genome-scale microarrays sample common SNPs with minor allele frequencies primarily in the 10-50% range, and successive generations will likely capture SNPs with lower minor allele frequencies. It should also be noted that it is the rare exception in which the SNP itself has been shown to have a causal pathologic mechanism, but the genetic loci of the SNP can implicate biologically relevant genes and pathways.

Adult height with its Gaussian distribution in the population is particularly well-suited for GWA studies. Beginning with identification of a common SNP in the *HMGA2* gene that conferred an estimated 0.4 cm increase in adult height in 2007 [48], the identification of genetic loci associated with height has been among the most successful of human GWA studies. Three independent groups reported their findings in 2008, escalating the total to over 40 loci associated with height [49-51]. More recently, the GIANT Consortium expanded their study to >180 thousand subjects, and reported at least 180 distinct loci that are associated with height [52]. The authors estimate that the SNP genotypes at these 180 loci explain approximately 10% of the variation of height in the population. It therefore follows that any single height allele explains only a small proportion of differences in height. Furthermore, identified SNPs with the greatest effect size are clustered with those with lower minor allele frequencies [53]. As 70-80% of heritability remains hidden, the predictive power from the current set of height GWA studies remains modest. On the contrary, these studies have been an unqualified success in providing biological insights to this field. The initial analyses by the GIANT Consortium described 21 of the loci to lie near OMIM skeletal or growth genes, with the majority of these known to be associated with skeletal dysplasias [52]. Taken further, Lui and co-workers used a combination of expression microarray of rodent growth plates and analyses of databases of human disease and mouse knockout phenotype to implicate 78 genes to growth plate function [54]. Many of the signaling pathways for these genes, for example PTHrP-IHH, BMP/TGF, and CNP, are not familiar to most clinical growth specialists, reinforcing the need for basic and clinical endocrinologists to maintain a continuous dialog. These genes may ultimately prove to function downstream of GH and IGF-1 at the growth plate, yet the mechanisms for convergence of these pathways have yet to be elucidated.

The list of genes identified by the height-GWA studies is also revealing in that it does not support many preconceived hypotheses on height. As discussed above, there is a rationale for the concept that common polymorphisms of genes of the GH–IGF-1 axis explain a significant proportion of the spectrum of height, and *GH1*, *GHSR*, *SOCS2*, *IGF1R*, and *IGF1BP2* are genes on the list that classically fit within this pathway (Figure 1). Yet conspicuous by their absences are the *GHR* and *IGF1* genes that would be consistent with the hormonal phenotype of primary IGF deficiency and partial GH resistance. These negative findings are consistent with a previous smaller candidate SNP study that surveyed common SNPs in 8 selected genes of the GH–IGF-1 axis in 2200 short or tall subjects and also failed to identify any significant associations [55]. Interestingly, a study in dogs had found the *IGF1* locus to be a major determinant in size, although clearly the selection of traits in breeding dogs and selective pressures during human evolution are difficult to equate [56]. As common variants near genes of the GH–IGF-1 axis constitute a small fraction of the total number of loci, which altogether explain only 10% of height heritability, it is safe to reject the long-held hypothesis that *common* polymorphisms of genes of the GH–IGF-1 axis play a substantial role in governing height.

Meanwhile, to address whether these common variants are impacting height in individuals with more extreme phenotypes, Chan *et al.* genotyped these SNPs in the 1214 subjects at the top and bottom 1.5 percentiles of two Scandinavian studies totaling over 78 thousand individuals [57]. While the pattern of alleles in the tall cohort matched the simulated proportions well, the height-associated SNP variants did not perform as well in the short cohort. In particular, the observed pattern in the shortest subset <0.25 percentile was significantly different than the simulated pattern. Therefore in the extremely short cohort, the current data suggest that models of rare genetic or non-genetic factors are more applicable than contributions from multiple common variants.

### Unbiased genetic technologies can identify molecular defects in unanticipated genes

In review, growth evaluations by pediatric endocrinologists have an inherent bias for ascertaining hormonal causes, particularly of the GH–IGF-1 axis, while the unbiased height GWA studies have revealed that known genes of the axis make up only a small fraction of the loci where common variants influence height. It therefore follows that rare single gene defects that present with poor growth will likely include a substantial proportion that lie in genes not commonly considered. Proportionally, common variants were exceedingly more prevalent in genes that function in the growth plate than those of the hormone axis [52,54], but it remains to be seen whether this will also be the case with rare variants. One would anticipate that in addition to overall short stature, single gene defects impacting the growth plate would present with features of skeletal dysplasia. Although abnormal body proportions can be evident on physical examination, many practitioners may find these differences difficult to recognize, as the phenotype may be subclinical. Therefore unbiased genome-wide technologies to identify rare defects should be considered as a potential tool to establish a molecular etiology for short stature.

Array-based technologies, including SNP genotyping arrays and comparative genomic hybridization, can detect copy number variations (CNV), either deletions or duplications, by comparing signal from a subject’s DNA to a reference standard [58]. Briefly, the subject and reference DNA are independently labeled with flurorophores of different colors and allowed to competitively hybridize to sequences sampling the entire genome on the test array. Deviation from the expected 1:1 ratio of subject and reference DNA at a given genetic loci would be suggestive of a potential deletion or duplication. The technique is commonly described as a genome-wide fluourescence in situ hybridization (FISH), where one does not dictate the genetic loci to be studied, although notably a directed FISH currently maintains greater sensitivity for small CNVs than array studies.

Array studies are being used increasingly in clinical settings concomitant with their decreasing costs. They are largely considered a first-tier test for evaluation of children with congenital structural anomalies or altered neurocognitive development, including those with autism spectrum disorders. In the latter, CNVs are identified in the range of 12-14%, providing a much higher diagnostic yield than standard karyotype [59]. Aside from case reports that detail identifying CNVs in individuals who had presented with poor growth, there have been a limited number of studies assessing CNVs on height. Dauber and colleagues investigated the impact of CNVs on growth by studying copy number burden in 4411 children with available height data who had a microarray study performed for other clinical indications [60]. They compared the CNV burden in the 415 subjects with height < -2 SDS, 196 with height > +2 SDS, and 3800 normal statured controls. Interestingly, they observed that the total CNV burden, both globally and restricted to genes, was significantly greater in the short, but not tall, children than the controls. Additional analysis revealed that deletions accounted for the difference in CNV burden in the short cohort, whereas there was no significant association with duplications. Given that common indications for attaining an array study are congenital anomalies and altered neurocognitive development, it is difficult to apply the findings of this study to a more general population.

Recently, the group of Zahnleiter *et al.* from Germany performed CNV analysis of 200 children with ISS (height < -2 SDS, average -2.75) and compared the results with 820 normal controls [61]. Even with stringent criteria for defining pathogenic CNVs (no overlap with CNVs of the control group, exclusion of strictly intronic or intergenic CNVs, either *de novo* or co-segregated with short stature if familial, and evidence in the literature for a growth phenotype at the locus), they identified a total of 10 deletions and 10 duplications in 20 families, ranging in size from 109 kb-14.2 Mb. 3 of these CNVs spanned one of the 180 height-associated SNPs [52], and several others spanned SNPs that demonstrated a trend for association but failed to meet the statistical threshold for a genome-wide study. The yield of 10% in this study (20 pathogenic CNVs in 200 children) is quite similar to that for autism spectrum disorders. If other groups confirmed a diagnostic yield in this range, there would be a reasonable argument that array studies should also be considered for all children with ISS. Presently they are not performed as common practice, and no groups have recommended their implementation in the diagnostic algorithm for poor growth.

Case reports of individuals presenting with a profound growth phenotype and subsequently identified to carry a single gene defect by unbiased sequencing are likewise increasingly common. Whereas in the past, positional cloning requiring multiple affected and unaffected individuals within a family was the primary modality to identify genetic lesions leading to a characteristic phenotype, investigators can now employ next-generation sequencing that allows for interrogation of DNA mutations in the exome or genome of a single presenting individual. A cursory literature search of the past 12 months reveals the use of whole exome sequencing in reports of short stature in individuals harboring mutations of *POC1A*, *NIN*, *CUL7*, *PIK3R1*, *KDM6A*, and *XYLT1*[62-69]. Notably, *PIK3R1* encodes the p85a regulatory subunit of phosphatidylinositol 3 kinase, that is a key downstream signaling molecule of insulin and IGF-1 [70], although the mechanism leading to poor growth in these short individuals has yet to be fully determined. Furthermore, two groups have employed candidate gene sequencing by first enriching for genetic regions of interest by using pre-designed biotinylated cRNA baits prior to next-generation sequencing [16,71]. This technique should increase specificity that an identified variant impacts growth, but sacrifices the potential to identify causal lesions in unanticipated genetic loci. Interestingly, among 192 children with short stature, Wang *et al.* identified 3 cases with known variants of *PTPN11* leading to undiagnosed Noonan syndrome and one probable pathogenic variant of *IGF1R*[71]. Similar to the array studies, the cost for performing next-generation sequencing is rapidly decreasing, with a $1000 genome test seemingly on the horizon. The initial experience of the clinical sequencing center at Baylor has recently been reported by Yang and colleagues [72]. Of the initial 250 consecutive cases with broad clinical phenotypic presentations, 62 were determined to have a mutated allele that was highly likely to be causative, providing optimism that this non-biased approach has sufficient yield to be included in diagnostic algorithms.

Aside from costs, there remain several issues to be addressed as these unbiased genetic technologies are offered more commonly [73]. Discriminating whether an identified abnormality is disease-causing or a benign variant is a major obstacle that has been addressed by the American College of Medical Genetics and Genomics [74]. Although there are multiple databases available to help classify an identified abnormality, they are by no means comprehensive. Some strategies previously mentioned in evaluating CNVs are directly applicable to sequence variants, including absence in unaffected individuals and co-segregation with the phenotype in families. For sequence data confined to exons, one can assess whether the predicted change in the encoded protein would likely alter its function, however a direct assessment of function of the gene product is usually desired. Mutations in gene regulatory regions outside of exons could also theoretically impact gene expression and present with a clinical phenotype, but the level of experimental evidence to have confidence in causation would have to be compelling. As such, whole exome sequencing is currently more practical than whole genome sequencing in detecting potentially causative genetic abnormalities. Next, whether identifying a genetic etiology for poor growth will impact clinical care, as envisioned by the allure of personalized medicine, has yet to be established. One could reasonably argue that identification of the etiology could increase suspicion for other features known to be associated with defects of a particular gene function. Data from common subsets of individuals formerly falling under the umbrella of ISS may reveal patterns that establish new paradigms for management. For example, recognizing that the most common mutation causing Noonan syndrome results in disruption of GH signaling has led some investigators to hypothesize that recombinant IGF-1 will achieve a better outcome than GH [40]. Lastly, the unbiased studies will certainly reveal new findings of an individual that were not anticipated or of unclear significance. How to manage this genetic information going forward is an important topic of public health [74].

Clinical experience with these unbiased technologies is rapidly accumulating. The accessibility of CNV analysis and whole exome sequencing to pediatric endocrinologists will at least partly be dictated by how third-party payers authorize reimbursement for these tests. As data about the yield for identifying pathogenic variants is still emerging, we propose that these diagnostic studies be seriously considered in the clinical evaluation in the five scenarios where the perceived yield is highest (Table 3). Given the greater experience with the array-based studies and their lower cost, it seems prudent to begin with CNV analysis before proceeding with whole-exome sequencing. As the field becomes more experienced with the studies, we would anticipate that the criteria for their use would evolve.

**Table 3:** Proposed criteria for unbiased genetic studies in ISS

**Criteria**	
●	Severe short stature, height < -3 SDS for CNV, <-4 SDS for whole exome sequencing;
●	Disproportionate short stature within family, height < -2 SDS from midparental target height;
●	Height < -2 SDS AND major congenital abnormalities or neurocognitive disorder;
●	Short stature segregating within the family in a dominant pattern;
●	History of consanguinity

### Epigenetic defects of key growth genes are a plausible mechanism for poor growth that is seldom evaluated

**Figure 2:**
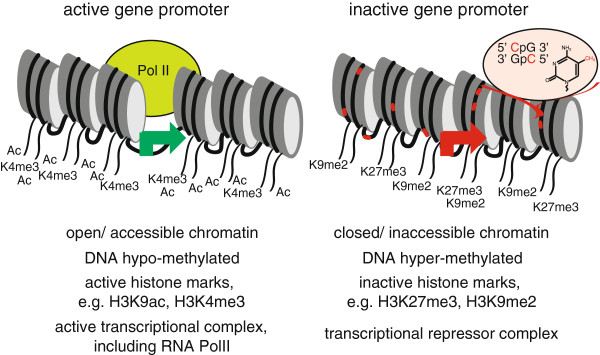
**The epigenetic context of gene regulatory elements can dictate gene expression.** Active and inactive gene promoters are distinguished by several epigenetic features including chromatin accessibility, DNA methylation, covalent modification of histone tails, and the types of transcriptional proteins in the vicinity. These epigenetic features are frequently dichotomous in the same gene in different tissues, and also may be different in the same gene within the same tissue of different individuals as an explanation for differences in gene profiles. By definition, epigenetic changes do not involve any differences in the underlying DNA sequence.

Mendelian genetic defects are the prototypical etiology that lead to changes in protein expression and function, but epigenetic changes represent another plausible mechanism that could give rise to changes in protein expression that manifest as a defined phenotype. Epigenetic changes are defined by 3 key characteristics, namely that they are stable, heritable and, and do not involve any changes of the DNA sequence [75]. Current research primarily focuses on two classes of epigenetic mechanisms, covalent modifications of histone tails and DNA methylation at cytosine residues in the context of cytosine followed by guanine (CpG). These influence gene expression at the level of chromatin, a term encompassing DNA with the associated histone proteins that allow its compaction. Characteristic patterns of chromatin at regulatory regions, e.g. promoters and enhancers, of actively transcribed genes include specific modifications of amino acid residues of histone tails and relative hypo-methylation at CpGs that occur together with increased accessibility and reduced compaction. Importantly, the chromatin landscape can be both gene- and tissue-specific, thereby allowing epigenetic changes to alter the transcriptional competence of a given genetic locus (Figure 2).

Epigenetic mechanisms have been proposed to serve as the basis for the developmental origins of adult disease (often termed the Barker) hypothesis [76]. By this model, adaptive responses to environmental influences in early life alter long-term risks for disease. For example, a perceived nutrient-poor environment that gives rise to poor intrauterine growth and SGA results in maladaptive changes in a postnatal environment of nutrient excess, such that individuals are predisposed to features of the metabolic syndrome including Type 2 diabetes. In a rodent model of IUGR induced by maternal uterine artery ligation that predisposes to diabetes, Park and colleagues demonstrated reduced expression of the pancreatic transcription factor encoded by *Pdx1* (homologous to *IPF1* in humans) in islet cells, with evidence of epigenetic silencing of the gene locus [77]. Interestingly, treatment with a GLP-1 analog in the first week of life reverts the epigenetic landscape at the *Pdx1* gene locus to normal and rescues the diabetes phenotype [78]. Epigenetic alterations in response to adverse intrauterine environments have been demonstrated in humans, for example in individuals prenatally exposed to the Dutch Hunger Winter of the Second World War [79]. Therefore there is a logical rationale that epigenetic alterations of key growth genes are a major mechanism for poor growth in IUGR/SGA.

In pediatric endocrinology, epigenetic mechanisms of disease pathogenesis are best illustrated with the complementary Beckwith-Wiedmann and Russell-Silver syndromes. It has recently been shown that changes in DNA methylation of differentially-methylated regions at the *IGF2/H19* locus are the most common identifiable molecular etiologies for these two syndromes [80]. In simplistic terms, changes from the normal methylation pattern lead to overexpression of *IGF2* in Beckwith-Wiedmann with overgrowth and underexpression of *IGF2* in Russell-Silver with growth retardation. To reiterate, traditional DNA sequencing at the locus does not reveal any differences of the nucleotide sequence, and specific assessment of the DNA methylation pattern is necessary to identify the change.

By analogy, one could imagine that epigenetic changes leading to diminished transcriptional competence at *GHR* or *IGF1* are a plausible mechanism for short stature in ISS, however there has yet to be any experimental evidence to support this hypothesis. Our laboratory has begun characterizing the chromatin landscape of the *Igf1* gene in rodents using a variety of techniques [81,82]. We have recently shown that the promoter of the gene is accessible in multiple tissues, but that defined enhancers are only accessible in liver, where the gene is most highly expressed [83]. This finding implies that molecular studies to assess the chromatin landscape of the gene in accessible cells or tissues, commonly peripheral mononuclear blood cells, may not be a representative surrogate for that of liver, the tissue of interest. Investigators should bear consideration of this before ruling out epigenetic changes as a potential contributing mechanism. Whether epigenetic changes of growth genes will be shown to be a major contributing mechanism to poor growth in SGA, and more generally in ISS, in the coming years will be of great interest.

## Conclusions

The majority of children who present for evaluation for short stature are not found to have an identified etiology and fall under the diagnosis of ISS. While rare single-gene defects impacting the GH–IGF-1 axis are well established as a mechanism for poor growth, recent studies refute the conventional hypothesis that common variants in these genes explain a significant proportion of short stature. Unbiased genetic technologies offer promise as tools to aid in diagnosis, but also introduce challenges that require careful consideration. Epigenetic alterations at specific genetic loci are another potential mechanism for poor growth that should be considered. As molecular etiologies for short stature are characterized more frequently in the coming years, the prospect of treatment plans individualized to the molecular etiology is intriguing.

## Abbreviations

CDGP: constitutional delay of growth and puberty; CNV: copy number variation; DNA: deoxyribonucleic acid; FDA: Federal Drug Administration; FISH: fluorescence in-situ hybridization; FSS: familial short stature; GH: growth hormone; GHBP: growth hormone binding protein; GWA: genome-wide association; IGF-1: insulin-like growth factor-1; ISS: idiopathic short stature; IUGR: intrauterine growth retardation; OMIM: Online Mendelian Inheritance of Man; RNA: ribonucleic acid; SDS: standard deviation score; SGA: small for gestational age; SNP: single nucleotide polymorphism; US: United States.

## Competing interests

The authors declare that they have no competing interests.

## Authors’ contributions

LAW made contributions to the writing, reviewing, and editing of the manuscript. DJC made substantial contributions in the conception, planning, implementation, writing, reviewing, and editing of the manuscript, and gave final approval of the version to be published. All authors have read and reviewed the final manuscript.

## Authors’ information

LAW is a first-year fellow and DJC is an Assistant Professor in Pediatric Endocrinology & Diabetes at the Icahn School of Medicine at Mount Sinai. The basic science interests of DJC include how epigenetics can influence gene expression in the IGF system.
